# Systematic Review of Usability Tests for Manual Wheelchairs: Enhancing Mobility and Reducing Shoulder Injuries in Individuals with Spinal Cord Injuries

**DOI:** 10.3390/jcm14093184

**Published:** 2025-05-04

**Authors:** Dongheon Kang, Jihyun Kim, Seon-Deok Eun, Jiyoung Park

**Affiliations:** 1 Assistive Technology Research Team for Independent Living, Rehabilitation Research Institute, National Rehabilitation Center, Seoul 01022, Republic of Korea; luxpooh@gmail.com (D.K.);; 2 Department of Safety and Health, Wonkwang University, Iksan 54538, Republic of Korea

**Keywords:** alternative propulsion technologies, manual wheelchair, shoulder pain, spinal cord injury, usability evaluation

## Abstract

**Background:** Manual wheelchairs (MWCs) are critical assistive devices for individuals with spinal cord injury (SCI) and other mobility impairments. However, inconsistencies exist in evaluating usability across different manual wheelchair designs. Usability evaluation methods are essential to ensure optimal design and function. **Methods:** A systematic review following PRISMA guidelines was conducted. Databases searched included PubMed, ScienceDirect, and DBpia. A comprehensive search was completed up to April 2024. Keywords combined concepts such as “spinal cord injury”, “manual wheelchair”, and “usability evaluation” using Boolean operators (AND, OR) and truncation strategies. **Results:** From 2134 initial records, 30 studies met the inclusion criteria. Studies included individuals with SCI as the primary population, but also incorporated able-bodied participants when necessary to simulate conditions not feasible for SCI users. Evaluation methods included objective assessments (e.g., kinematics, kinetics, electromyography) and subjective measures (e.g., System Usability Scale, user interviews). **Conclusions:** This review highlights methodological trends in MWC usability testing and identifies key metrics to guide future research and design improvements. While the primary focus was on individuals with SCI, studies involving healthy participants were included where ethically or practically justified.

## 1. Introduction

Manual wheelchair (MWC) use is often regarded as a physically demanding and inefficient form of mobility, particularly for individuals with spinal cord injury (SCI), due to its association with repetitive upper limb strain and long-term musculoskeletal complications [1,2]. The upper limbs, which are essential for propulsion via hand rims, are highly vulnerable to discomfort and injury. Numerous studies have reported a high prevalence of shoulder pain and overuse injuries among MWC users [3,4,5], especially among individuals with SCI, resulting in decreased independence and quality of life [6]. These complications not only impair mobility but also restrict other daily living activities involving upper limb function.

In response to these challenges, various alternative propulsion mechanisms have been developed. For example, pushrim-activated power-assist wheelchairs (PAPAWs) incorporate motorized components that support propulsion through battery-powered systems [7], targeting users with SCI, upper limb pain, or general muscle weakness [8]. However, such models typically increase the weight of the wheelchair and limit maneuverability. Lever-propelled wheelchairs provide biomechanically favorable propulsion movements and are effective for outdoor use, yet their restricted hand path and reduced efficiency pose limitations [9,10,11,12]. However, similar to PAPAWs, they restrict hand movement to a fixed trajectory and reportedly exhibit lower propulsion efficiency [13,14,15].

To address these issues comprehensively, usability evaluation has emerged as a central criterion for developing and refining manual wheelchair systems. According to ISO 9241-11 [16], usability is “the extent to which specified users can use a product to achieve specified goals with effectiveness, efficiency, and satisfaction in a specified context of use”. This review adopts that framework to evaluate usability testing methodologies in MWCs, emphasizing user-centered outcomes.

While individuals with SCI represent this review’s core population of interest, several included studies used able-bodied participants. These participants were typically involved in simulations where experimental risks, ethical limitations, or technical constraints made direct inclusion of SCI users difficult. Therefore, their inclusion was justified in cases where the usability dimension, such as propulsion effort or device handling, could be objectively assessed through analogous tasks.

This study aims to systematically categorize and analyze existing usability evaluation methods for manual wheelchairs, examining their application across different wheelchair designs and user groups. By reviewing both conventional and novel MWC types, the study seeks to (1) identify key usability evaluation tools, (2) uncover practical user demands based on experimental outcomes, and (3) provide strategic guidance for the future development of effective, user-responsive manual wheelchairs, particularly for individuals with SCI. Although this review primarily focused on individuals with spinal cord injury (SCI), several studies involving able-bodied participants were included when safety concerns or feasibility issues precluded direct testing with SCI individuals. These inclusions were carefully justified to ensure relevance and applicability to the SCI population.

## 2. Materials and Methods

### 2.1. Study Design

This systematic review follows the Preferred Reporting Items for Systematic Reviews and Meta-Analyses (PRISMA) guidelines.

The PICOS framework was used during the study design phase to establish the criteria for selecting relevant studies (Table 1). P (participants) was individuals with SCI; C (comparison) was the control group for randomized controlled trials; and S (study design) was the experimental studies. Among groups of individuals that use MWC, individuals with SCI were selected as the study population because of their reliance on wheelchairs for daily activities and mobility. Moreover, prolonged wheelchair use in this population imposes repetitive loads on the upper limbs, often resulting in shoulder joint damage, pain, and various associated conditions [17]. I (intervention) was not applicable to this study, as the focus was to review the results of usability testing for MWCs. O (outcome) was not specified to allow for a broad examination of the methods and results of the usability testing.

### 2.2. Literature Search

Academic journal publications (excluding theses and abstracts) were included in this systematic review. Searches were conducted over seven days in two rounds: from March 27 to April 1, 2024 (first round) and April 24 to 26, 2024 (second round), to ensure comprehensive coverage of recently published studies. The literature was retrieved using PubMed and ScienceDirect for international articles, and DBpia for Korean language publications. A comprehensive search was conducted across PubMed, ScienceDirect, and DBpia up to April 2024. No additional updated search was performed after this period.

Keyword combinations included “spinal cord injury”, “manual wheelchair”, and “usability evaluation”, with Boolean operators and truncation strategies applied. The Advanced Research option was used for ScienceDirect, with all keywords entered into the “Find articles with these terms” field and re-entered under “Title, abstract, or author-specified keywords”. The Advanced Search feature was employed for PubMed with all keywords entered simultaneously. On DBpia, a combination of one or more keywords was used to yield diverse results, including the following:

(1) “Spinal cord, injury, manual, wheelchair, usability”;

(2) “Manual, wheelchair, usability”.

While the original PICOS criteria focused on individuals with SCI, several studies involving able-bodied participants were also included where appropriate, in order to simulate usability conditions that were not feasible or ethically suitable for direct testing with SCI participants.

### 2.3. Literature Selection and Classification

**Figure 1:**
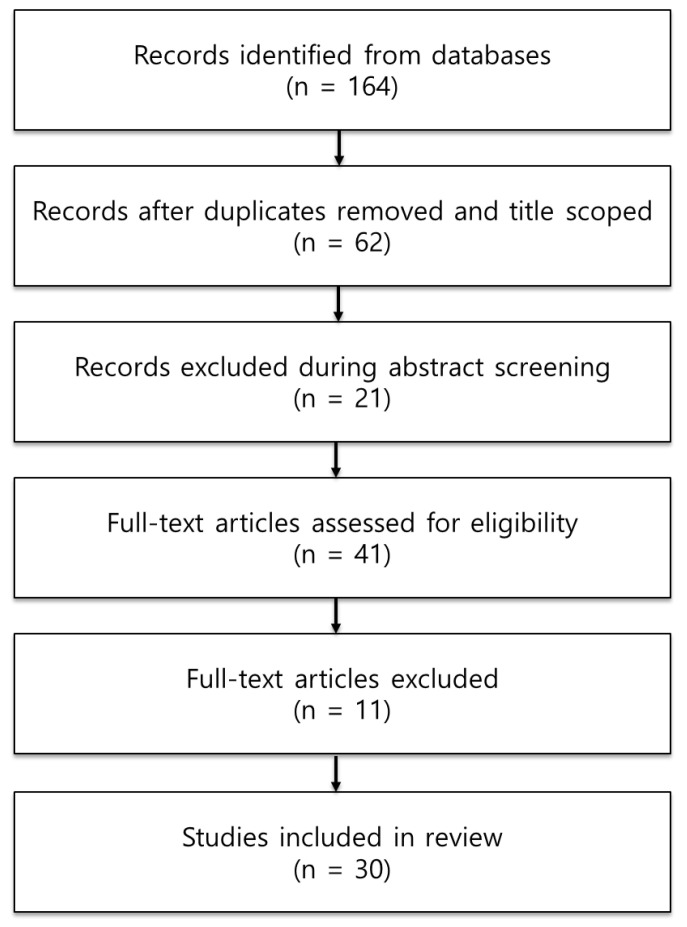
PRISMA 2020 flow diagram for new systematic reviews, including searches of databases and registers alone.

The selection and classification process adhered to the PRISMA 2020 flow diagram for new systematic reviews, which includes only databases and registers (Figure 1). During the first screening stage, the titles of retrieved articles were reviewed, and duplicates were removed. Abstracts were then evaluated to determine their relevance based on the PICOS framework. In the second screening stage, abstracts were reassessed, and articles were excluded based on the following criteria: (1) studies categorized as literature reviews or meta-analyses; (2) studies not involving individuals with SCI (e.g., usability tests using tasks unsafe for individuals with SCI or involving participants without wheelchair experience); and (3) studies not focused on MWCs (e.g., studies on powered wheelchairs, wheelchair-accessible vehicles, or wheelchair kiosks). After the screening, full texts were reviewed, and the following exclusion criteria were applied: (1) unavailable full text, (2) literature reviews and meta-analyses, (3) studies that did not involve individuals with SCI (excluding some studies), and (4) studies that did not involve MWCs. Automation tools were not used for the screening process. One researcher independently performed the classifications, and a second researcher comprehensively reviewed the results for appropriateness and completeness. A total of 30 studies were selected, comprising one Korean study (Table 2) and 29 international studies (Table 3).

To enhance the methodological rigor of the review, all 30 included studies were appraised using a modified version of the Critical Appraisal Skills Program (CASP) checklist. Each study was evaluated across five domains: (1) clarity of study objectives, (2) adequacy of participant description, (3) validity of outcome measures, (4) transparency of usability testing procedures, and (5) appropriateness of statistical analysis. These criteria categorized studies as high, moderate, or low quality (Supplementary Table S1).

To improve the transparency of the selection process, we clarified the rationale for inclusion and exclusion based on usability testing approaches. Specifically, studies were included if they conducted structured usability evaluations involving either subjective user assessments (e.g., SUS, QUEST 2.0) or objective physiological/kinematic measurements (e.g., EMG, SMARTWheel). Studies were excluded if they (1) did not include empirical usability testing, (2) evaluated only general satisfaction or preferences without linking them to performance metrics, or (3) examined automated or powered wheelchair systems without any manual propulsion component. Additionally, while the primary focus was on individuals with SCI, some studies involving able-bodied participants were included. These cases were justified when simulations were used to replicate usability conditions, such as high-speed propulsion or high-risk maneuvering, and were not feasible or ethical for direct testing with individuals with SCI.

### 2.4. Data Extraction

A full-text review was conducted for the 30 selected studies, and detailed data were extracted according to the study objectives and PICOS framework. The extracted data were organized into the following categories: primary author (year of publication), participants, MWC characteristics, experimental protocol, outcome measures and results, and conclusion.

The participants in the selected studies were predominantly individuals with SCI, as defined by the PICOS framework. However, a few studies included participants with other conditions, such as multiple sclerosis. Additionally, some studies involved individuals without disabilities when tasks in the usability tests were unsuitable or unsafe for individuals with SCI or when participants lacked prior wheelchair experience. Some of these studies were included to provide a comprehensive analysis of various findings related to MWC usability that align with the objectives of the review.

For the experimental protocol, details such as the experimental conditions for MWC use, utilization of motion analysis systems, and inclusion of electromyography (EMG) sensors were separately categorized and documented. The sequence of the experimental procedures was recorded based on the tasks performed by the participants.

## 3. Results

### 3.1. Participants

The demographic data of the participants included various SCI-related characteristics, including duration of injury, level and grade of impairment, complete/incomplete injury, and location of the spinal cord lesion. Most participants with SCI had prior experience with MWCs or had used MWCs as their primary mobility aid. Among four studies that involved individuals without disabilities [15,30,36,41], two included participants with no prior wheelchair experience. Pain assessments or questions were administered in 18 studies, with 7 utilizing the Wheelchair User’s Shoulder Pain Index (WUSPI) or its variant, PC-WUSPI, at baseline.

### 3.2. Characteristics of MWCs Used in the Experiment

The MWCs used in the experiments fell into three broad categories: (1) MWCs previously used by the participants, (2) PAPAWs, and (3) MWCs with new features or propulsion mechanisms. Seven studies compared standard MWCs with PAPAWs, which are equipped with DC motors installed in the rear wheel hubs to supplement propulsion based on the user’s pushrim input. PAPAWs have shown great potential for improving MWC usability among individuals with SCI [8,37,46]. Six studies evaluated MWCs with new features or propulsion systems, including (1) two-speed MWCs utilizing planetary gear trains [19,20], (2) MWCs equipped with two types of anti-rollback devices [22], (3) MWCs featuring a pulley–cable propulsion system utilizing a rowing gesture [30], (4) MWCs employing an ergonomic hand drive mechanism (EHDM) with a cam-pawl and ratchet system [32], and (5) MWCs with 12 configurations combining three seat heights and four antero-posterior axle positions [8]. Three of the six studies (50%) focused on developing and evaluating the usability of new propulsion systems for MWCs, highlighting the importance of developing advanced propulsion mechanisms to improve usability and prevent injuries.

In 12 studies, specialized wheels (SMARTWheel, Intelliwheel, and Propulsiometer) were attached to MWCs to measure kinetic data, including force and torque. Additionally, three studies utilized strain-gauge force transducers; one used a Hall sensor, and another employed a six-degree-of-freedom force transducer. Three studies [31,36,42] analyzed stroke patterns rather than the mechanical properties of MWCs.

### 3.3. Methods of Evaluation

The experimental conditions (driving conditions) were categorized as follows: (1) flat surfaces, such as tiles, carpets, and track fields, as well as ramps and curbs (6 studies); (2) rollers and ergometers (dynamometers) (16 studies); (3) treadmills (5 studies); (4) course driving (2 studies); and (5) wheelchair skill tests (WSTs) (1 study). For the two studies on course driving, Guillon et al. [27] developed indoor and outdoor driving courses to evaluate the participants’ wheelchair-handling abilities under diverse terrain conditions, whereas Algood et al. [40] designed a multi-obstacle course that incorporated indoor and outdoor environments to assess the practicality of wheelchair use. The details of the courses are provided in Table 4. Wheelchair driving speeds were categorized into three settings: (1) self-selected speed, (2) predefined steady-state speeds, and (3) maximal acceleration conditions (acceleration trials). More than half of the studies (16 studies) adopted the self-selected speed approach. The conditions in the predefined steady-state speed included 0.56 m/s, 0.83 m/s (3 km/h), 0.9 m/s (2 mph), and 1.8 m/s (4 mph). One study [36] used a metronome to ensure steady-state driving. The usability evaluation methods employed in each study—including evaluation type, tool, and setting—are summarized in Table 4. This table categorizes studies by subjective/objective design, assessment tools, and evaluation conditions. Driving course details—including indoor and outdoor conditions used in usability assessments—are listed in Table 5. These environments helped simulate real-world use conditions.

### 3.4. Outcome Measures and Results

Seventeen studies employed motion analysis systems, whereas six utilized EMG sensors. Five studies combined motion analysis systems and EMG sensors in their experiments. The anatomical landmarks and sensor placement locations based on these systems are summarized in Table 5. Studies that collected data from either the dominant or non-dominant side of participants provided specific justifications for their selection: The dominant side was chosen to simulate a painful region [26], whereas the non-dominant side was selected owing to its potential impact on curb-climbing tasks [21]. Seven studies included physiological measurements, including heart rate, blood pressure, oxygen consumption and ventilation, VO_2_ efficiency, and energy cost (L/m). Six studies, including a Korean study, employed the usability test tools for participants’ subjective assessments. The tools included SUS, Borg RPE Scale, a custom-designed functional rating scale [22], QUEST 2.0 (Quebec User Evaluation of Satisfaction with assistive Technology), satisfaction evaluations using the visual analog scale [27], obstacle completion difficulty and ergonomic evaluation using the visual analog scale [40], WST, WheelConP-SF, WheelCon-M-SF, and NASA-TLX. Although most studies measured and statistically analyzed variables, such as velocity, range of motion (ROM), force, and moment to determine outcomes, some uniquely focused on spinal curvature, shoulder pathology, and nerve conduction data to explore their associations with physical changes related to MWC use. Additional outcome measures included the number of stops or collisions during tasks, time to complete tasks, self-reported discomfort or pain, and number of participants requiring assistance to complete tasks. Table 6 provides a side-by-side comparison of subjective and objective usability outcomes. It summarizes the tools used and key results, revealing consistencies and divergences in user perception versus biomechanical data. The sensor placement locations for motion and biomechanical analysis are presented in Table 7, which identifies anatomical and wheelchair landmarks used for data collection.

To enhance interpretability, we synthesized the results across propulsion technology types and summarized their respective strengths and limitations in a new comparative table (Table 8). This table integrates both objective and subjective findings, helping to clarify which technologies demonstrated superior usability outcomes in terms of biomechanical performance and user satisfaction. Such classification aids clinicians and designers in identifying the most promising design directions.

## 4. Discussion

This systematic review aimed to categorize and evaluate the usability assessment methods employed in manual wheelchair (MWC) studies across diverse user groups, focusing on individuals with spinal cord injuries (SCI). The primary objective was to determine how usability is evaluated—subjectively and objectively—and to identify trends in testing environments, propulsion mechanisms, and outcome metrics.

The reviewed studies employed a variety of usability evaluation methods. As summarized in Table 4 and Table 6, subjective evaluation tools, such as SUS, QUEST 2.0, and NASA-TLX, were used in a minority of studies. In contrast, objective assessments using motion capture, EMG, and kinetic sensors (e.g., SMARTWheel) were more frequently applied. However, only a few studies integrated both approaches. The limited use of comprehensive tools suggests a gap in evaluating usability in a manner that reflects both biomechanical performance and user satisfaction.

Alternative propulsion systems—such as gear-driven, lever-propelled, and pulley–cable mechanisms—have demonstrated potential for reducing physical strain during wheelchair use. Jahanian et al. [2,19,20] and Cavallone et al. [30] reported that these technologies improved biomechanical efficiency without compromising function. These findings align with Table 6, illustrating variations in subjective and objective outcomes depending on the propulsion type. While motion analysis and kinetic data remain central in MWC studies, user-centered evaluation remains underutilized. Only 6 of the 30 studies included subjective assessment tools, and only two incorporated in-depth interviews. As shown in Table 6, user satisfaction data—when collected—often diverged from biomechanical results, highlighting the necessity of dual-perspective evaluations. Training and adaptation protocols also emerged as a key usability factor. Mattie et al. [47] and Kim et al. [18] emphasized the role of structured learning in reducing user discomfort and increasing confidence. Incorporating such procedures in usability studies can reduce outcome variability and improve ecological validity. Moreover, the sensor placement patterns detailed in Table 7 underscore the importance of methodological consistency in motion and EMG assessments. Standardized anatomical and wheelchair landmarks can facilitate cross-study comparisons and better quantify performance outcomes.

From a clinical perspective, one of the most significant practical findings of this systematic review is that alternative propulsion technologies—such as PAPAWs, lever-drive systems, and pulley–cable mechanisms—consistently demonstrate biomechanical advantages that reduce shoulder joint stress and muscular effort. These findings have clear implications for rehabilitation specialists, occupational therapists, and assistive technology professionals tasked with recommending or customizing wheelchairs for long-term users. For instance, PAPAWs and planetary gear systems showed consistent reductions in peak propulsion forces, suggesting that these systems may delay the onset of shoulder pain or injury in high-risk users with SCI. Clinicians should consider integrating such technologies into early-stage mobility training programs to prevent musculoskeletal complications. Additionally, pulley–cable systems—by activating antagonist muscle groups—may offer therapeutic benefits for maintaining upper extremity balance and coordination. Furthermore, the heterogeneity in usability evaluation methods across studies highlights the need for developing standardized clinical protocols that integrate both biomechanical data and user feedback. This dual-approach framework would enable evidence-based selection and customization of MWCs aligned with users’ functional profiles, thereby optimizing both safety and long-term satisfaction. In practice, usability test outcomes should not remain confined to laboratory or engineering settings. Instead, they should inform clinical decision-making processes—particularly in tailoring training interventions, evaluating readiness for independent mobility, and selecting propulsion mechanisms suited to patients’ physical limitations and environmental demands.

In sum, the usability evaluation of MWCs should integrate objective performance data with subjective user experiences. This would ensure that technological improvements translate into real-world benefits for individuals with SCI. Future studies should adopt multidimensional evaluation frameworks that include (1) validated, user-centered tools, (2) structured adaptation protocols, and (3) consistent sensor methodology. Such approaches will improve the interpretability of MWC usability data and enhance the design of assistive devices that are truly responsive to user needs.

Based on the findings of this review, several specific recommendations can be made to guide future wheelchair design and usability research. First, alternative propulsion systems should be developed with careful attention to minimizing joint-specific strain, especially at the shoulder and wrist, as biomechanical analyses have shown that these joints are most vulnerable to overuse injuries. For example, pulley–cable or lever-based systems may help distribute load more evenly across muscle groups. Second, designers should consider integrating adjustable configurations—such as seat height, axle position, and propulsion handle orientation—based on users’ body size, level of injury, and propulsion technique. Studies in this review demonstrated that even subtle configuration differences can significantly affect propulsion efficiency and comfort. Third, usability testing protocols should include both subjective (e.g., SUS, QUEST 2.0, NASA-TLX) and objective (e.g., EMG, kinematic/kinetic) evaluations to ensure that designs align with both user perception and biomechanical safety. Fourth, future research should prioritize long-term usability studies that simulate real-world conditions, including obstacle navigation and transfers, which better reflect daily user experiences. Finally, interdisciplinary collaboration between engineers, clinicians, and end-users is critical to ensure that design innovations are functional, safe, and aligned with the diverse needs of manual wheelchair users. These recommendations offer a more actionable roadmap for researchers and practitioners seeking to improve manual wheelchair usability across varied user populations.

This review is novel in systematically integrating both objective biomechanical evaluations and subjective user-centered assessments across diverse propulsion technologies. It advances the field by clarifying which usability measures are most informative for both research and clinical practice. Furthermore, by addressing multiple user populations, the findings offer more generalizable insights applicable beyond individuals with SCI alone. For clinicians, the findings suggest evidence-based recommendations for wheelchair selection and configuration, aiming to reduce upper extremity strain and improve mobility outcomes.

## 5. Conclusions

This systematic review categorized and analyzed 30 studies that assessed the usability of manual wheelchairs (MWCs) across diverse user groups, including, but not limited to, individuals with spinal cord injuries (SCI). The review found that while a range of alternative propulsion systems—such as lever-crank, gear-driven, and pulley–cable mechanisms—have been developed to reduce upper limb strain, usability testing remains inconsistent across studies.

Most studies focused on objective data such as motion analysis or electromyography, while relatively few incorporated subjective tools or user feedback through interviews or questionnaires. As a result, the practical utility and perceived satisfaction with new wheelchair designs remain insufficiently explored. Furthermore, the expression of “effectiveness” varied widely across studies, and no standardized metrics were used to compare usability outcomes.

Future studies should adopt integrated evaluation frameworks that combine objective biomechanical data with subjective user experience measures to enhance the real-world impact of MWC innovations. Establishing standardized usability evaluation protocols is critical to ensuring that assistive technologies are functionally beneficial, accepted, and valued by users daily.

This review mapped the landscape of manual wheelchair usability evaluation methods, identifying critical gaps and proposing standardized approaches for future studies. While the primary focus remained on individuals with SCI, findings highlight the need to validate usability outcomes across broader user groups. Integrating objective biomechanical metrics and subjective user satisfaction measures will be crucial for advancing evidence-based wheelchair design and clinical decision-making.

## Figures and Tables

**Table 1:** PICOS.

Participants	Individuals with SCI
Intervention	None
Comparison	(Comparison group)
Outcome	-
Study design	Experimental studies

SCI, spinal cord injury.

**Table 2:** Data extracted from the Korean literature.

	First Author (Year)	Participants	MWC Characteristics	Experimental Protocol	Outcome Measures and Results	Conclusion
1	J. Kim (2018) [18]	Three individuals with SCI (selected based on disability level) T10, T4–T5, and C3–C4 injuries	(1) Participant 1 (T10) Wheelchair: MWC + electric bike module Propulsion: Pushrim + bike module handle (2) Participant 2 (T4–T5) Wheelchair: MWC Propulsion: Pushrim (3) Participant 3 (C3–C4) Wheelchair: MWC Propulsion: Push handle for caregiver	(1) Baseline interview collected basic information, outdoor wheelchair use, and activity details. (2) Observation scenarios of “Preparation, Movement, Task, Completion” were analyzed for “user–product, user–caregiver, user–environment” interactions. Meals and walking (Participant 1, T10), shopping (Participant 2, T4–T5), hospital visits and meals (Participant 3, C3–C4) (3) Post-interview: Unique aspects of MWC use observed in the “observation” and satisfaction evaluation (SUS)	(1) User–product interaction Participant 1: Tilting of the wheelchair with the attached module; module handle positioned at a distance; overall increased length Participant 2: Difficulty carrying items Participant 3: Primary propulsion is handled by the caregiver; need for improvements to reduce the caregiver’s physical effort during transfers or movement (2) User–caregiver interaction Participant 1: Module attachment improved independence, communication, and confidence Participant 2: Adjustments needed for backrest and push-handle height Participant 3: Armrests and backrests need improvements for high-level cervical SCI (3) User–environment interaction: Participant 1: Elevator and table usability issues; no outdoor parking for detached modules Participant 2: Need for accessible parking; discomfort with checkout counter height and location in stores Participant 3: Excessive physical effort required from the caregiver (4) SUS Participant 1: SUS 87.5, Acceptable, B, Excellent Participant 2: SUS 92.5, Acceptable, A, Excellent Participant 3: SUS 95, Acceptable, A, Excellent	(1) Reasons for choosing MWCs (economic reasons, adaptability to body size for posture maintenance, compactness, and lightweight) (2) Preference for electric bike modules or hybrid wheelchairs to expand activity range (3) Lower SUS scores noted for learnability-related items

MWC: Manual Wheelchair; SCI: Spinal Cord Injury; SUS: System Usability Scale.

**Table 3:** Data extracted from the international literature.

	First Author (Year)	Participants	MWC Characteristics	Experimental Protocol	Outcome Measures & Results	Conclusion
1	O. Jahanian (2022) [19]	7, SCI, paraplegia MWC use as the primary mode of mobility. Minimum of 6 months’ experience with an MWC. Having the ability to perform independent transfers Shoulder pain assessment using WUSPI	Personal standard MWC equipped with Intelliwheels standard gear (1:1) and low gear (1.5:1) ∙Two-speed planetary gear train	•Condition: tile, carpet, ramp (1) Acclimation time: 15–30 min (2) Propelled their wheelchairs on a 10 m tiled level floor, 8 m carpeted level floor, and 2.5 m ramp with a 4.8 deg slope (3) Both standard/low gear conditions in random order (4) Start from a resting position up to their self-selected normal speed	•In all ground conditions (tile, carpet, ramp) •In the low gear condition (1) Significant decrease in propulsion speed, peak resultant force, peak propulsive moment, peak rate of rise in the resultant force, and normalized integrated moment (2) Significant increase in the number of stroke cycles (normalized to distance)	Although a higher number of stroke cycles are required to travel a given distance in a low gear than the standard gear condition, the low gear condition might be less demanding for the upper extremity
2	O. Jahanian (2022) [20]	7, SCI, paraplegia Use an MWC as the primary mode of mobility Have a minimum of 6 months’ experience as an MWC Have the ability to perform independent transfers Shoulder pain assessment was administered by WUSPI	MWC standard gear (1:1) and low gear (1.5:1)	•Motion analysis •Surface EMG •Condition: carpet (1) Acclimation time: 15–30 min (2) Propelled their wheelchairs 8 m carpeted level floor (3) Both standard/low gear conditions in random order (4) Start from a resting position up to their self-selected normal speed	•In the low gear condition (1) Significant decrease in propulsion speed, stroke distance, peak hand-rim resultant force/moment, peak glenohumeral inferior force/flexion moment, peak/integrated muscle activity of the anterior deltoid and pectoralis major, and perception of effort (2) No significant difference in normalized integrated muscle activity (muscle activity per stroke distance)	Usage of geared wheels may be beneficial for wheelchair users to enhance independent mobility in their homes and communities while decreasing their shoulder demands
3	Lalumiere et al. (2013) [21]	15, SCI Used an MWC as their primary means of mobility (>4 h/day) No or minimal pain in the shoulder, which did not limit their ability to ascend curbs (max = 12 cm) (mean WUSPI = 0.11 ± 0.15/10)	Personal standard MWC equipped with Smartwheels (instrumented wheels)	•Motion analysis •Surface EMG •Conditions: **curbs** (4, 8, and 12 cm high) from a starting line set 3 m before the curb •Three curb heights could not be randomly tested (1) Familiarization period (2) Ascend a curb while propelling at their self-selected natural velocity (caster pop▶ rear-wheel ascent▶post-ascent)	•Non-dominant upper extremity (U/E) (likely to limit the performance of the curb ascent tasks) •Between the curb heights (4▶8▶12) (1) The greatest effort was generated by shoulder flexors, internal rotators, and elbow flexors (2) Significant increase in movement excursion, net joint moments, and muscular utilization ratio (MUR) (3) No significant difference in the total duration of the caster pop/rear-wheel ascent mainly around the shoulder joint	(1) Ascending a curb requires substantial U/E efforts, especially for the shoulder flexors, internal rotators, and elbow flexors (2) U/E efforts progressively augment as the height of the curb increases despite movement strategy modifications (e.g., increase forward trunk flexion) (3) Importance of the highly structured training program when learning wheelchair curb ascent and the task-specific strength training programs based on U/E demand for curb ascent
4	S.L. Deems-Dluhy (2017) [22]	12, SCI, AIS, traumatic/non-traumatic use of an MWC as the primary form of mobility for a minimum of 3 months before the study, Self-reported perceived pain (NPRS, SCI-specific numeric pain rating scale) Upper/Lower Extremity Motor Scores (UEMS/LEMS)	Standard MWC fitted with WARDs (Wheelchair Anti-Rollback Device, prototype “wheel” and “brake”)	•Physiological data •Condition: ramp (1) Training: propelling the wheelchair to ascend the ramp, controlling backward tipping, manipulating, and WARDs to switch between neutral and engaged positions (2) Testing: ascending a 1:12 grade, 7.3 m ramp with a 90 deg turn in three ramp ascent test conditions (“wheel”, “brake”, “no-WARD”; order was randomized) propelled up the ramp at their self-selected pace engaging/disengaging the WARDs to stop as they needed to rest	(1) Per trial (three trials per condition) Perceived level of exertion (Borg RPE scale): “brake” > “wheel” > ”no-WARD” Number of stops: “brake” > “wheel” > ”no-WARD” (=0) Time taken to finish: “brake” > “wheel” > ”no-WARD” Self-reported pain/discomfort: wrist pain (n = 2), back pain (n = 1) (2) Per condition Custom-designed functional rating scale (similar to patient-specific functional scale): “wheel” is more difficult to manipulate (engage/disengage) during ramp ascent than “brake” Satisfaction (QUEST2.0): “wheel” is more compact (dimension) than “brake” (3) Before the start and after the finish Heart rate: no significant difference Blood pressure: no significant difference	(1) Users that could benefit most from the version of the investigated WARD prototypes were those with lower levels of spinal impairment (2) The device design needs improvement for easier reach and manipulation
5	A. Gil-Agudo (2010) [23]	51, SCI, C6–L3, ASIA A/B Daily users of MWC reported no upper extremity pain in the past Group 1 (G1, n = 12): C6 tetraplegia Group 2 (G2, n = 8): C7 tetraplegia Group 3 (G3, n = 17): paraplegia, T1–T10 Group 4 (G4, n = 14): paraplegia, T11~L3	Standard adjustable wheelchair with the Smartwheels	•Conditions: treadmill •Power output was determined by a drag test •All participants: right-hand dominant (1) Adaptation period: 2 min (2) Propelled the wheelchair at 3 km/h for 1 min	•External power output of the treadmill during the drag test = 18.3 W •Shoulder - peak force: G1, G2 > G3, G4 - maximum moment on the x-axis - adduction moment: G1, G2 > G3, G4 •Elbow - vertical force: G1 > G3, G4 - medial force: G1 > G3, G4 - anterior force: G3 > G1 - peak adductor moment: tetraplegia > paraplegia •Wrist - vertical force: G1 > G4 - peak cubital deviation, pronation, and palmar flexion moments of force: G3, G4 > G1, G2 - wrist moment: constant	(1) Patients with tetraplegia have upper limb impairment and can successfully complete the task of MWC propulsion with relevant adaptions in the kinetic pattern (2) Increasing superior joint forces in the shoulder, elbow, and wrist Adduction moment in the shoulder Constancy of the moments of force on the wrist may increase the risk of developing upper limb overuse injuries in the tetraplegia population
6	C.J. Newsam (1999) [24]	69, complete SCI 17, low paraplegia (T10-L3) 19, high paraplegia (T1-T9) 16, C7 tetraplegia 17, C6 tetraplegia Daily users of a lightweight wheelchair are free of upper extremity pain and do not indicate a history of upper extremity dysfunction prior to SCI	MWC (rigid frame and lightweight; three strain-gauge force transducers are instrumented on the right pushrim)	•Motion analysis •Conditions: stationary ergometer (1) Familiarization period: a few minutes/on the laboratory’s level surface (2) Accustomed period: a few minutes/on the stationary ergometer (3) Propel the test wheelchair at a normal, comfortable pace•Propulsion cycle (PC) = push (positive wheel torque > 0.4 Nm) +recovery (when the wheel torque was below 0.4 Nm and lasted until the onset of the next push phase) Push sub-phase: Initial Contact (IC)▶early TC (Top-Center)▶Late TC recovery sub-phase: Hand Off (HO)▶Follow-Thru (FT)▶Arm-Return (AR)▶Push Preparation (PP)	•Eight joint motions of the U/E and trunk (1) Humeral Mean peak anterior plane: 13~22°, FT Maximal elevation: paraplegia (55° and 54°) > C6 (45°) Minimal elevation: 21°, FT and AR Rotation: no significant differences between the groups (2) Elbow Timing of peak elbow flexion earlier: paraplegia (17.7% and 15.1%) > C6 (36.9%) Forearm pronation (supination): C6 tetraplegia had significantly less pronation than all the other groups (3) Wrist Wrist flexion/extension: motion patterns for wrist flexion/extension were remarkably different between the paraplegia and tetraplegia groups Wrist ulnar/radial deviation: initial contact was made with 7° of radial deviation for all groups, except the C6 (0°) group (4) Trunk Extension (total excursion of trunk motion): C6 (11°) > all other groups (5~6°)	(1) Kinematic patterns of most joints were similar between the four groups of participants with SCI. (2) The strategy of hand contact with the pushrim was different for participants with C6 tetraplegia who required greater wrist extension and less forearm pronation to substitute the ulnar aspect of the palm for absent finger grasp
7	K. Kulig (2001) [25]	69, complete SCI 17, low paraplegia (T10-L3) 19, high paraplegia (T1-T9) 16, C7 tetraplegia 17, C6 tetraplegia No upper extremity pain in the past	MWC (a lightweight rigid frame with an adjustable footrest and seat back height; three strain-gauge force transducers were instrumented on the right pushrim)	•Motion analysis •Conditions: stationary ergometer (1) Familiarization period: propel the wheelchair on the ergometer for several minutes (2) Propel the test wheelchair at a normal, comfortable self-selected speed •No voice motivation or encouragement was given to the participants during data collection	(1) Mean self-selected propulsion velocity Low paraplegia: 90.7 m/min High paraplegia: 83.4 m/min C7 tetraplegia: 66.5 m/min C6 tetraplegia: 47.0 m/min (2) Shoulder joint moments - no significant differences (3) Superior push force C7 (21.4 N) > C6 (9.3 N) > high paraplegia (7.3 N)	(1) Propelling a wheelchair imposes comparable, moderate loads on the shoulder joint during both phases of wheelchair propulsion, despite the relatively slower velocities of persons in the tetraplegic groups. (2) Since propulsion is a cyclic activity, this continuous demand may contribute to localized muscle fatigue
8	U. Arnet (2024) [26]	16, SCI, chronic tetraplegia (C4–C8) MWC dependency for ADL, either with/without supportive propulsion LPG (low pain group, n = 6): PC-WUSPI < 7.5 MPG (moderate pain group, n = 6): 7.5 ≤ PC-WUSPI ≤ 35.5 HPG (high pain group, n = 4): PC-WUSPI ≥ 35.5	Smartwheels Side where shoulder pain was highest Dominant side if the user did not report pain	•Conditions: motorized treadmill (1) Familiarization period (2) Performed two pre-defined speed/power output conditions for 1 min each in a randomized order 0.56 m/s, 10 W 0.83 m/s, 15 W	(1) Spatio-temporal characteristics Push angle: HPG > LPG, MPG > LPG Absolute recovery time: LPG > HPG Relative propulsion time: MPG > LPG (2) Force application Maximal medial force: MPG > LPG, MPG > HPG FEF_mean (Fraction of Effective Force): HPG (40.7 ± 5.8%) was significantly different from LPG (35.2 ± 14.7%) and MPG (40.8 ± 15.9%)	(1) Persons with high levels of shoulder pain applied propulsion force more effectively (with a lower medial force component) and over a longer push angle, thus shortening the recovery time (2) Persons with high levels of shoulder pain (HPG) propel their wheelchair more optimally than those with LPG to MPG
9	B. Guillon (2015) [27]	52, SCI, ASIA A/B Ability to use both arms to propel an MWC over several hundred feet Ability to ambulate unassisted	Conventional MWC 3 PAPAWs (E-motion, servomatic A, and servomatic B)	•Physiological data •Conditions: ① Dynamometer roller (n = 10) resistance 15 W/30 W 1.8 m/s ② Indoor and outdoor courses (n = 46) ③ Transfer themselves and their wheelchairs into and out of their car (n = 10)	(1) Dynamometer VO_2_ (Oxygen consumption per unit time): 3 PAPAWs decreased with 15 W/30 W compared with conventional MWC Heart rate: 3 PAPAWs decreased with 15 W/30 W compared with conventional MWC (2) Outdoor driving Maximal heart rate: MWC > B = EM > A Completion time: EM > MWC > A > B Handrim push frequency: MWC > EB > A > B Satisfaction (VAS): A = B > MWC > EM (3) Indoor driving Completion time: EM > B > A > MWC Handrim push frequency: EM > A = B > MWC Number of collisions: EM > A > B > MWC Satisfaction (VAS): MWC > A > B > EM (4) Transfer into and out of the car the number of participants who required help: MWC (n = 0), Servomatic (n = 3), E-motion (n = 4) completion time Satisfaction (VAS): MWC > 3-PAPAWs	(1) Compared with E-motion, the 2-Servomatic PAPAWs (A/B) were easier to use outdoors (2) Difficulty transferring into/out of the car was similarly increased with all three PAPAWs
10	R. Price (2007) [28]	13, SCI Complete/incomplete SCI below T1 Using an MWC as a primary means of mobility (>40 h/wk) No history of upper limb fracture or dislocation No history of arm and shoulder pain as a result of syrinx	MWC-Smartwheel (non-dominant side)	•Motion analysis •Conditions: passive dual-roller system MAC: maximal acceleration SSS: steady-state, self-selected speed (1) Acclimation time (2) Propel under two conditions: MAC and SSS (3) SSS: data capture was performed such that at a minimum, the last 20 s of the trial were at a steady-state pace in the judgment of an investigator monitoring (4) MAC: instructed via the computer’s display to accelerate maximally for 5 s, brake and rest for 5 s, accelerate maximally again for 5 s, and then coast for the remainder of the trial	•Shoulder power: MAC > SSS •Elbow/wrist power: unchanged •Peak shoulder power fraction: MAC > SSS •Peak elbow power fraction: SSS > MAC •Peak wrist power fraction: SSS > MAC	(1) Power at the shoulder was larger than that at other points (2) Higher joint power, present under MAC, may predispose MWC users to injury, particularly at the shoulder
11	M.S. Nash (2008) [29]	18, SCI, C5-L1 12, paraplegia 6, tetraplegia Used an MWC as their primary means of mobility Having confirmed shoulder pain (WUSPI)	Participant’s customary wheelchair Same wheelchair retrofitted with PAPAWs (E-motion)	•Physiological data •Conditions: stationary platform with rollers •Five testing sessions (1 + 4) on nonconsecutive days within 2 weeks (1) One session (familiarization period): 30 m (customary wheelchair), 30 m (PAPAWs) (2) Two sessions: consisted of unresisted, steady-state wheelchair locomotion for 6 min at the greatest attainable speed (3) Two sessions: consisted of graded wheelchair locomotion for 12 min at the greatest attainable speed (resistance was applied to the rollers in 1 kg increments every 2 min using a calibrated force meter)	•6 m steady-state test sessions (1) Oxygen consumption (VO_2_): customary > PAPAW (2) Distance (m): PAPAW > customary, paraplegia > tetraplegia (3) Energy cost (L/m): customary > PAPAW (4) Rating of perceived exertion (Borg RPE scale): time was the only significant effect •12 m resistance-graded test sessions (1) Oxygen consumption (VO_2_): paraplegia (significant increases at each time point), tetraplegia (no significant difference) (2) Distance (m): PAPAW > customary, paraplegia > tetraplegia (3) Energy cost (L/m): customary > PAPAW (4) Rating of perceived exertion (Borg RPE scale): customary > PAPAW	Use of PAPAWs by persons with paraplegia and tetraplegia having shoulder pain significantly lowers energy cost responses and perceived exertion compared with MWC propulsion while significantly increasing the distanced propelled
12	P. Cavallone (2022) [30]	7, able-bodied Non-expert in the use of wheelchairs None of the participants reported musculoskeletal disorders that could affect upper limb movements	Hand wheelchair. Q Pulley–cable propulsion systems (rowing gesture) pushrim propulsion systems •Two hall sensors (the angular velocity of the wheels, each mounted on the wheelchair frame beside the left/right rear wheels)	•Motion analysis •Surface EMG (dominant side) •Conditions: roller test bench (1) Familiarization session: 5 min, practicing with both propulsion systems at variable speeds (2) Propel the wheelchair using the pushrim and the pulley–cable systems, at low and high speeds, four trials each lasting roughly 2 min, ensuring at least 30 movement cycles per trial (performed the tasks at a consistent cadence) •Low speed: self-selected, a comfortable speed, as if they had to move around normally •High speed: twice as fast as the comfortable (low) speed condition	(1) Wheelchair and joint kinematics Duration of cycles: pulley > pushrim, low speed > high speed Linear displacements: high > low Wheelchair displacements: pulley–cable propulsion is three times greater than pushrim propulsion Joint kinematics: pulley–cable propulsion demanded a greater range of elbow, not shoulder flexion/extension (2) Onset of muscle excitation and silencing pushrim: muscles were excited early in recovery and silenced at mid-drive, except for the anterior deltoid Pulley–cable: The opposite was observed, resulting in significantly different onset values for all muscles represented (3) Muscle excitation and duty cycle movement speed: no effect Pulley–cable: ① Greater duty cycle: posterior deltoid, supraspinatus ② Smaller duty cycle: pectoralis major	(1) Opposite muscle groups were excited when comparing the two mechanisms for wheelchair propulsions. (2) Participants can move faster without overloading the shoulder muscle with the hand wheelchair. Q.
13	S.L. Walford (2021) [31]	SCI, paraplegia WUSPI scores were under 12 at the baseline subset1 (n = 60): baseline (pain-free) →18 months (no pain) →36 months (pain, n = 19; no pain, n = 41) subset2 (n = 25): baseline (pain-free) →18 months (shoulder pain) →36 months (chronic, n = 12; pain resolved, n = 13)	MWC-Smartwheel (kinetic) (right handrim) •Hand patterns (1) AR (arcing) (2) SL (single-loop): expected to correlate with shoulder pain owing to its higher muscle demand and associated propulsion mechanics (3) DL (double-loop): under-rim patterns (4) SC (semi-circular): under-rim patterns	•Motion analysis (kinematic) •Biodex System 3 Pro dynamometer (strength, peak maximal isometric torque) •Three time points (baseline, 18 months, 36 months) •Conditions: stationary ergometer •Task: propelled their own wheelchair with the resistance set to emulate overground propulsion on a tile surface at their fastest comfortable speed (1) Trial of fast propulsion of up to 30 s for accommodation (2) One to two minutes of rest to prevent fatigue (3) Last 10 s data collection of a 15 s trial	(1) NRT (Net Radial Thickness) Positive: over-rim pattern Negative: under-rim pattern (2) TRT (Total Radial Thickness) Smaller: hand pattern stays near the handrim Greater: hand pattern moves farther away from the handrim (1) Subset 1 Pain group use a more over-rim pattern No significant differences between the pain and no pain groups for either NRT or TRT at any time point (2) Subset 2 No differences in NRT or TRT between individuals whose shoulder pain continued at 18 months (chronic pain) or resolved at 36 months (pain resolved)	(1) Hand pattern used during fast propulsion is not correlated with shoulder pain (2) More over-rim hand patterns may indicate weaker shoulder adductors
14	B.A. Cloud (2017) [32]	21, SCI of spinal cord disease, C6/7–L2 ≥1 y of MWC use Independent MWC use Active shoulder range of motion within limits needed to perform propulsion during the study procedures Completed the WUSPI (18 participants reported some level of pain on the WUSPI)	MWC—Two different seat dump angles with a vertical backrest 0° 14° (SmartWheel—unpublished data)	•Fiberoptic sensors (spinal curvature) •Electromagnetic sensors system (shoulder motion) Thorax: over the manubrium Scapula: at the superior surface of the acromion Humerus: via a thermoplastic cuff •Conditions: custom rollers (1) Familiarization: completed several propulsion cycles (2) Complete at least three cycles of propulsion at a self-selected speed (if more cycles were completed, only the first three cycles were used for data analysis)	(1) Spinal curvature full group: significantly less lordosis in the 14° Low-level SCI: on average, less lordosis in the 14° High-level SCI: no significant differences in lordosis or kyphosis (2) Shoulder complex kinematics full group: ① Internal rotation was higher in the 14° seat dump angle condition at SP (start of push) and MP (mid-push) ② Upward/downward rotation value was larger in the 14° at SP and MP Low-level SCI: no significant differences High-level SCI: at SP, MP, MR (mid-recovery)/except for EP (end of push) ① Higher values of internal rotation in the 14° ② Increased values of upward/downward rotation	(1) In those with high-level SCI, the adaptation at the scapula appeared to be strongest (2) In those with low-level SCI, the change in spinal curvature was strongest (3) Different seat angles do not appear to directly influence the risk of subacromial impingement because no differences were observed in the glenohumeral joint
15	L.A. Zukowski (2014) [33]	12, SCI 7, paraplegia 5, tetraplegia Full-time MWC users	(1) EHDM: Ergonomic Hand Drive Mechanism (used a cam pawl and ratchet mechanism that grabs onto the tire tread for forward propulsion and releases during the recovery phase) (2) CMW: Conventional MWC	•Physiological data •Conditions: semicircular track (99.3 m) (1) Propelled the EHDM and pushrims until they were comfortable with the operation of the chair in either mode (2) Propel around a 99.3 m, semicircular track making only gradual, rounded right-hand turns for 3.5 min at a steady, self-selected pace	(1) Velocity: CMW > EHDM (2) Distance traveled: CMW > EHDM (3) Number of pushes: no difference (4) VO_2_ consumption: no difference (5) VO_2_ efficiency: CMW > EHDM (6) Heart rate: no difference	(1) Performance and efficiency were sacrificed with the EHDM (2) Modifications to the EHDM (e.g., addition of gearing) could rectify the performance and efficiency decrements while maintaining similar metabolic costs
16	R.E. Cowan (2008) [34]	128, SCI (Case series)	MWC-Smartwheel	•Conditions: tile surface, low pile carpet, ramp (1) Tile and carpet Began from a stationary position, accelerated to a comfortable self-selected velocity, and pushed for a maximum of 10 s/10 m/end of the surface (2) Ramp From level ground directly in front of the ramp, and with casters touching the ramp threshold, users propelled up the full length until reaching a platform	(1) Self-selected velocity: ramp < carpet < tile (2) Peak resultant forces: ramp > carpet > tile (3) Push frequency: unchanged (4) Stroke length: unchanged	Investigators have described a protocol to evaluate MWC propulsion in clinics, and have presented preliminary data generated from this evaluation protocol
17	L. Lighthall-Haubert (2009) [35]	14, SCI, ASIA A/B tetraplegia C6 (n = 5) C7 (n = 9) Pushed an MWC for at least 50% of their locomotion	PAPAW (Quickie Xtender) MWC (standard pushrim)	•Motion analysis •Surface EMG •Dynamometer (shoulder strength) •Conditions: ergometer •Fixed order: WC▶PAPAW (1) Standard pushrim WC propulsion ① Accommodation: 3–5 min, on the ergometer ② Self-selected free velocity, self-selected fast velocity without additional load applied to the ergometer rollers, simulating propulsion over level ground ③ Elevated with wooden blocks and resistance added to the ergometer rollers to simulate an 8% grade (or a 4% grade if the participants were unable to sustain propulsion with 8% resistance grade) (2) PAPAW propulsion ① Accommodation: 5–8 min, on the ergometer ② Attempt to match propulsion speed, ±5%, to that recorded during the similar matched standard pushrim WC propulsion condition (free, fast, and graded) with verbal feedback from the tester and visual feedback from a speedometer	(1) PAPAW propulsion matched to the standard wheelchair ① Velocity-matched fast propulsion: statistically but not clinically significantly reduced ② Velocity-matched graded propulsion: significantly faster than WC ③ Cycle length: significantly greater ④ Cadence: significantly reduced ⑤ EMG median intensities: WC > PAPAW peak intensities: WC > PAPAW Duration of activity: generally reduced (2) Self-selected free PAPAW propulsion ① Velocity: more than twice as fast as self-selected free WC propulsion ② Cycle length: significantly greater than both free and fast WC propulsion ③ Cadence: similar to free WC, less than fast WC ④ EMG peak: similar to free WC, significantly less than fast WC Duration: similar to free WC (despite propelling at greater than twice the speed of WC)	For participants with complete tetraplegia, push phase shoulder muscle activity was decreased in the PAPAW compared with standard pushrim WC, indicating a reduction in demands when propelling a PAPAW
18	B. Bickelhaupt (2018) [36]	18, able-bodied 17, right hand dominant SC patterns group P patterns group	• Propulsion patterns (1) SC (semicircular propulsion technique): favored by experienced wheelchair users (2) P (pump propulsion technique): energetically more efficient in inexperienced wheelchair users	•Dynamometer (U/E strength) •Condition: wheelchair treadmill (1) Familiarization period: 30 m, practice stroke technique, SC or P (2) Propel the wheelchair at a rate of approximately 1 stroke/s, using a metronome (60 bpm) as a guide •Borg RPE scores collected at 2 min intervals starting at zero minutes and continuing until 10 min of continuous wheelchair propulsion •Pre- and post-test dynamometer readings for bilateral elbow and shoulder extension	(1) Shoulder muscle fatigue (Borg RPE) Time: statistical significance within subject effect Propulsion methods: no significant difference (2) Strength (dynamometer): the effect of technique on postexercise strength was not statistically significant for shoulder/elbow measures of strength	SC wheelchair propulsion pattern appears to be more fatiguing to shoulder muscles than the P propulsion pattern
19	S.D. Algood (2004) [37]	15, cervical level SCI Full-time MWC users for at least 1 y with tetraplegia Used ultra-lightweight wheelchairs as their primary means of mobility Free from pressure ulcers and shoulder pain Have no history of cardiopulmonary disease	PAPAW traditional MWC	•Physiological data •Motion analysis •Condition: wheelchair dynamometer •Three different resistances: slight, 10 W/flat tiled floor, moderate, 12 W / flat carpet, high, 14 W / uphill •Maintain a speed of 0.9 m/s (1) Acclimation time: 5 min (2) Propelled both their own MWC and a PAPAW on a computer-controlled wheelchair dynamometer during 3 min each trial	(1) Variations in mean velocity 10 W: do not differ significantly 12 W: do not differ significantly 14 W: PAPAW > MWC (2) Metabolic energy consumption, oxygen consumption and ventilation: a significance difference between MWC and PAPAW Mean heart rate: significantly reduced when participants used the PAPAW during the high resistance trial (3) Stroke frequency 10 W: significantly reduced 12 W: significantly reduced 14 W: no significant difference (4) ROM: significantly lower when using the PAPAW 10 W: shoulder flexion/extension, internal/external rotation, horizontal flexion and extension; wrist ulnar and radial deviation 12 W: shoulder flexion/extension, internal/external rotation, horizontal flexion/extension; forearm supination/pronation, ulnar and radial deviation 14 W: for all joint movements except for shoulder abduction and adduction	For participants with tetraplegia, PAPAWs reduce the energy demands, stroke frequency, and overall joint ROM compared with traditional manual MWC propulsion
20	J.L. Mercer (2006) [38]	33, SCI, paraplegia 29, right-hand dominant SCI below the level of T1 that occurred after the age of 18 years Used an MWC as their primary means of mobility No upper limb pain prohibited them from propelling an MWC	MWC-Smartwheel (Each participants propelled his or her own wheelchair during testing)	•Motion analysis •Condition: wheelchair dynamometer (2 independent rollers, one for each wheel) 0.9 m/s (2 mph) 1.8 m/s (4 mph) (1) Propel at two speeds: 0.9 m/s and 1.8 m/s (2 and 4 mph) (2) After each participants reached a steady-state speed, data were collected for 20 s	•Shoulder pathology (1) Physical examination: 10 participants (30%) reported discomfort during one or more clinical tests during the physical exam (2) Magnetic Resonance Imaging (MRI): non-dominant side: participants exhibited mild and moderate abnormalities for all eight MRI measures •Shoulder biomechanics (1) Higher posterior force, lateral force, and extension moment during propulsion: more likely to exhibit coracoacromial ligament edema (2) Larger lateral forces, abduction moments: more likely to have coracoacromial ligament thickening (3) Higher superior forces and internal rotation moments at the shoulder: associated with increased signs of shoulder pathology during the physical examination	Specific joint forces and moments were related to measures of shoulder pathology
21	M.L. Boninger (2004) [39]	35, SCI case series Traumatic SCI at the T2 or below that occurred more than 1 year previously Use MWC for mobility Have no history of trauma to the shoulder or wrist	MWC-Smartwheels	•Motion analysis •Conditions: wheelchair dynamometer - 0.9 m/s (2 mph) - 1.8 m/s (4 mph) (1) Acclimation period (2) Propel at two different speeds: 0.9 m/s (2 mph) and 1.8 m/s (4 mph) (3) Once the participants reached the desired steady-state speed, both kinematic and kinetic data were collected for 20 s	•Nerve conduction studies (median/ulnar nerve) (1) Wrist kinematics mean peak wrist flexion: less motion at a faster speed (2) Wrist kinematics and nerve function ulnar/median latencies and wrist kinematics: no significant correlation ulnar/median motor amplitudes and wrist motion: higher ranges of wrist motion were associated with increased median and ulnar motor amplitudes (3) Wrist kinematics and pushrim kinematics ulnar/median nerve amplitudes and wrist ROM: positive correlations Wrist flexion/extension ROM and stroke frequency: negative correlations Wrist flexion/extension ROM and resultant force: negative correlations	(1) Subjects using a greater ROM showed better nerve function than subjects propelling with a smaller ROM (2) Participants using a larger ROM used less force and fewer strokes to propel their wheelchairs at a given speed (3) It is possible that long, smooth strokes may benefit nerve health in MWC users
22	S.D. Algood (2005) [40]	15, cervical-level SCI, tetraplegia Full-time MWC users for at least 3 months Free from pressure ulcers and shoulder pain that would prevent them from propelling an MWC	PAPAW traditional MWC	•Physiological data (heart rate) •Conditions: ADL course (obstacle course) (1) Opportunity to familiarize themselves with the course before testing (2) Propel their own MWCs and a PAPAW 3 times (total trials, 6) over an ADL simulation course •After the 1st, 3rd, 4th, and 6th trials, participants were asked to fill out survey parts 1/2, portion of the survey that queried aspects of the wheelchair and the ADL driving course •Questions specific to PAPAW (survey part (3) were administered after each participant’s last trial using PAPAW	•Participant survey and tester rating survey (1) Questions pertaining to the difficulty of completing the obstacles (VAS) between 1st and 3rd trials: 8 obstacles as significantly easier to complete (carpet, up ramp, bump, curb cut, toilet, bathroom sink, turning on the kitchen faucet, and bus docking space) (2) Questions pertaining to the ergonomics of the wheelchairs (VAS) after 3rd trial: PAPAW was significantly easier to propel and more comfortable (3) Questions specifically related to the PAPAW •Heart rate: MWC > PAPAW •Time to complete course mean time to complete the course: no significant difference	PAPAWs have the potential to improve functional capabilities during certain ADLs, especially when propelling up ramps, over uneven surfaces, and over thick carpet
23	Y.S. Yang (2006) [41]	14, unimpaired participants (able-bodied) No previous history of upper extremity pain or abdominal/back injuries	MWC-Smartwheels (a three-dimensional force and torque sensing wheel)	•Motion analysis •Surface EMG •Condition: dynamometer - 2 steady-state speeds of 0.9 and 1.8 m/s - Acceleration from rest to their maximum speed (1) Propelled at two steady-state speeds: SLOW (0.9 m/s), FAST (1.8 m/s) (2) Completed one acceleration trial (ACC) that involved a quick acceleration to their fastest possible propulsion speed, and maintaining the speed for a 6 s period •Instructed to push without leaning against the backrest (in order to elicit the highest level of trunk muscle recruitment) •Propulsion cycle push phase: early push, late push recovery phase: follow-through, hand return, pre-push	(1) Push phase sEMG SLOW: dominant activity on three back muscles (LT, IL, MU) and one abdominal muscle (EO) FAST and ACC: abdominal muscles (RA, IO, EO) increased their onset intensity level as the back muscles (LT, IL, MU) Overall: The onset intensity of the back muscles across three speed conditions was significantly higher than abdominal muscle intensity during the push phase (2) Recovery phase sEMG SLOW: The dominant muscle is the same that was active during the push phase (LT, MU, EO)/IL remained inactive FAST and ACC: The intensity of abdominal and back muscle activity increased Overall: The activity of the back muscle groups across all three speed conditions was significantly larger than the abdominal muscle activity during the recovery phase	(1) Both back muscle (LT, MU, IL) and abdominal muscle (RA, IO, EO) groups illustrated the highest intensity during the pre-push and early push stages of the propulsion cycle (2) These two muscle groups co-contracted to provide sufficient trunk stability for the propulsion tasks
24	W.M. Richter (2007) [42]	26, SCI Low-level (T12-L5), mid-level (T7–11), high-level (T2–6) Full-time users of an MWC Comfortable propelling a wheelchair for periods of 2 min Have full use of their upper extremity Have no medical conditions that could be aggravated by wheelchair propulsion	MWC-Propulsiometer • Stroke patterns (1) Arcing (2) Semi-circular (3) Single-looping (SLOP) (4) Double-looping (DLOP)	•Motion analysis •Condition: treadmill (1) Practiced pushing on the treadmill at a range of speeds (2) Chose a comfortable speed for level, 3°, and 6° grades. (3) Pushed for 35 pushes on level, 30 pushes on the 3° grade, and 25 pushes on the 6° grade, all at their self-selected speeds	(1) Handrim biomechanics (as a grade was increased) Speed: avg 63% decreased Peak force: avg 218% increased Push angle: 25.5% decreased Push frequency (cadence): 21.6% decreased Power output: 6°(41.9 W) > 3°(39.2 W) > level (10.2 W) (2) Stroke patterns The number of participants who used each of the three patterns was fairly balanced for level propulsion Once participants began pushing uphill (3°, 6°), many who were not using the arcing pattern (DLOP, SLOP) began to use it No participant used the semi-circular pattern	(1) Wheelchair users will change their stroke pattern to accommodate their propulsion environment (2) There were no biomechanic advantages or disadvantages for any pattern
25	J.L. Collinger (2008) [43]	61, SCI below T1 case series 56, right-hand dominant (non-dominant side data were analyzed) Paraplegia Used an MWC as their primary means of mobility Had no upper-limb pain that prohibited them from propelling an MWC Asked two questions about shoulder pain	MWC-Smartwheel	•Motion analysis •Condition: wheelchair dynamometer (2 independent rollers, resistance = tile surface) (1) Acclimation time (2) Three speed trials Self-selected comfortable pace 0.9 m/s (2 mph) 1.8 m/s (4 mph) •Real-time speed and direction feedback were displayed on a monitor in front of participants during the trials	(1) Influence of shoulder pain No significant differences in propulsion biomechanics between participants who reported shoulder pain in the last month and those who reported no shoulder pain No significant differences in propulsion biomechanics between persons who did and did not experience pain while propelling (2) Demographics and biomechanics The primary demographic variable that affected shoulder forces was body weight (3) Biomechanics and propulsion speed Mean self-selected speed: 1.09 m/s All shoulder forces and moments followed the same pattern observed for the mean velocities, with the lowest values occurring for 0.9 m/s and the highest values resulting during the 1.8 m/s trial The largest directional force component: posterior force The largest directional moment: the internal rotation moment (4) Kinetic/kinematic timing For all speed conditions, the peak resultant force at the shoulder occurred during the first half of the push phase; the same is true for the most directional force and moments Peak resultant force occurred later as speed increased	(1) Significant increases in shoulder joint loading were observed as propulsion velocity increased (2) Pain did not affect shoulder biomechanics during propulsion (3) Body weight was the primary demographic variable related to glenohumeral joint loading (4) Peak shoulder joint loading occurs when the arm is extended and internally rotated, which may leave the shoulder at risk of injury
26	R.A. Cooper (2001) [8]	9, SCI, T2–12 1, multiple sclerosis (MS) full-time, community-dwelling MWC users	PAPAW participants’ MWC	•Physiological data •Condition: wheelchair dynamometer normal: ③ 0.9 m/s, 10 W ① 1.8 m/s, 25 W slight: ④ 0.9 m/s, 12 W ② 1.8 m/s, 30 W moderate: ⑤ 0.9 m/s, 14 W •Phase 2: metabolic energy consumption (1) Acclimation time: 5 min (2) Propel their own chair and a PAPAW on a computer-controlled wheelchair dynamometer •Phase 3: ADL evaluation (1) Propelling over a standardized ADL course 3 times (2) Completed a portion of the survey (Likert-type scale from 0 to 10) after completing the 1st trial and the remainder after finishing the 3rd trial	•Phase 2: metabolic energy consumption Oxygen consumption: MWC > PAPAW Oxygen ventilation: PAPAW > MWC at ②, ⑤ Heart rate: MWC > PAPAW at ②, ⑤ •Phase 3: ADL evaluation No significant differences identified for the physical strain and completion time values of each trial between the wheelchairs PAPAW had the highest mean ergonomic ratings No significant difference in the number of stroke events between the wheelchairs (MWC = 47, PAPAW = 51)	PAPAWs may provide MWCs with a less stressful means of mobility with few adaptations to the vehicle or home environment
27	N. Louis (2010) [44]	10, paraplegia, wheelchair experienced 10, able-bodied, wheelchair non-experienced Right-handed Free of prior injury or pain related to the upper limb Male	MWC with 12 configurations (3 different seat heights, 4 different anteroposterior axle positions)	•Motion analysis •Surface EMG •Condition: roller ergometer (1) 12 wheelchair configurations were selected in the range allowed by the custom-built experimental chair (2) Accommodation: a few seconds (3) Propel a MWC at a self-selected speed and 15 consecutive propulsion cycles were recorded	(1) Timing of propulsion phases Early push (EP) is significantly longer for the paraplegic group than for the able-bodied group No significant difference in push percentage over propulsion cycle between the two groups (2) Experience in wheelchair Muscle activation and recruitment are different between the two groups (PAPAW > able-bodied) (3) Seat height Early push: biceps, brachii and pectoralis major EMGi decreased Late push: biceps brachii peak decreased Recovery: deltoid posterior peak and EMGi and biceps brachii peak and EMGi decreased (4) Anterior–posterior axle position Early push (axle forward, increased): deltoid anterior peak and EMGi, deltoid posterior peak and EMGi, trapezius peak and EMGi, triceps brachii peak and EMGi, pectoralis major EMGi Late push (axle forward, increased): deltoid anterior peak recovery (axle forward, increased): trapezius peak and EMGi, biceps brachii peak	(1) Future studies investigating wheelchair propulsion with a focus on upper limb overuse injuries linked to propulsion should limit participant recruitment to individuals with paraplegia who are experienced wheelchair users (2) Effects of settings are not the same during early-push, late-push, recovery, or the complete cycle.
28	D.J.J. Bregman (2009) [15]	5, able-bodied (AB) 8, paraplegia (PP) 3, tetraplegia (TP) No current shoulder problems Experienced wheelchair users who use a wheelchair on a daily basis At least 1 yr post-injury	Kinetics-instrumented wheelchair (6-degrees-of-freedom force transducer built into the right wheel)	•Motion analysis •Delft shoulder and elbow model •Condition: treadmill (1) Accustomed period: a few minutes (2) Propel 1 m at a constant velocity of 0.83 m/s (which approximates low-load daily wheelchair propulsion)	(1) Kinematics Average propulsion cycle duration: 1.34 (0.27) s, no significant difference between the three groups Push phase: 51.7% of the entire propulsion cycle (2) Kinetics Mean force onto the right handrim: 18.8 N, no significant difference between the three groups Tangential component of the propulsion force: 11.7 N FEF: 63.2% •Delft shoulder and elbow model (1) Physiological cost Square root of physiological cost: 32.2▶42.0 No significant difference between 3 groups (2) Net joint moments Peak anteflexion moment: 3.4 Nm, end of the push phase Peak retroflexion moment: 5.1 Nm, half through the push phase Peak adduction moment: 3.4 Nm Peak internal rotation moment: 3.9 Nm in the tangential force condition; the peak anteflexion moment was significantly higher, and shifted more toward the end of the push phase compared to the experimental condition (3) Energy and power Both produced energy and dissipated energy of all muscles were significantly higher in the tangential force condition than in the experimental force condition (4) Glenohumeral constraint and contact force The glenohumeral contact force was found to be at its peak in the middle of the push phase in both force conditions (tangential, experimental) The glenohumeral contact force increased in the tangential force condition during the entire push phase The mean peak glenohumeral contact force was significantly higher in the tangential force condition	(1) Tangential propulsion force in MWC propulsion appears to be less efficient owing to the occurrence of co-contractions around the elbow and a higher production of power around the shoulder joint (2) Higher production of power around the shoulder joint makes tangential force propulsion not only less efficient, but also more straining for the shoulder
29	J. Mattie (2020) [45]	11, SCI 7, paraplegia 4, tetraplegia 1, multiple sclerosis (MS) Using an MWC as their primary mobility device Adequate trunk control (as measured by their ability to sit on a hard surface with only the support of their arms) Adequate U/E functioning to safely operate the Nino (measured by their ability to grip the steering tiller)	MWC Nino	•Participants completed the Wheelchair Skill Test (WST) for MWC users, version 4.1, both for their MWC and the Nino^®^ •The WST comprises 32 skills, but skills 13, 23, 26–32 were omitted (e.g., negotiating 15 cm curbs, wheelie-related tasks) •1st session (MWC) (1) WheelCon-P-SF (confidence with MWC use): represented participants’ anticipated confidence levels, primarily based on their initial perceptions of the Nino (2) WST for MWC (3) WheelCon-M-SF (confidence with power wheelchair use) for MWC (4) NASA-TLX (task-load demand) for MWC 5) Completed Nino training protocol (1–6 levels) •2nd session (Nino, 2 weeks later) (1) Completed Nino training protocol (1–12 levels) (2) WheelCon-P-SF for Nino (3) WST for Nino (4) WheelCon-M-SF for Nino (5) NASA-TLX for Nino (6) Qualitative interview (30 m): A semi-structured interview guide guided by UTAUT (Unified Theory of Acceptance and Use of Technology)	(1) WST: Nino > MWC (2) WheelCon (confidence): increased significantly after completing the Nino training Significantly more confident when using their own MWC than with Nino, measured post-training (3) NASA-TLX: Nino > MWC (4) Qualitative data Performance expectancy: would best be suited for outdoor use; not a functional device that could replace their MWC Effort expectancy: required precise movements to maneuver in small spaces, and overall, users commented on the increased mental and physical challenge required, with most users reporting difficulty while braking Social influence: Nino would have a positive impact on social interactions Facilitating conditions: Users wished there was increased adaptability and customizability of the Nino/needs a strong lift or assistance to bring into the car/the financial burden of purchasing the device	The Nino is unlikely to be suitable as a functional replacement for an individual’s MWC

AR, arching; CMW, conventional manual wheelchair; DL, double-loop; EHDM, ergonomic hand drive mechanism; EMG, electromyography; EP, end of push; MAC, maximal acceleration; MP, mid-push; MR, mid-recovery; MWC, manual wheelchair; NRT, net radial thickness; PAPAW, power-assist pushrim-activated wheelchair; RPE, rating of perceived exertion; SC, semi-circular; SCI, spinal cord injury; SL, single-loop; SP, start of push; SSS, steady-state, self-selected speed; TRT, total radial thickness; VO_2_, oxygen consumption; WUSPI, wheelchair user’s shoulder pain index.

**Table 4:** Usability evaluation methods by study.

Study	Method Type	Evaluation Tool	Driving Condition	Outcome Measure Type
Jahanian (2022) [2]	Objective	SMARTWheel	Flat surface	Kinetic
Lalumiere (2013) [21]	Objective	EMG + Motion	Curbs	Kinematic + EMG
Cavallone (2022) [30]	Objective	Motion + EMG	Roller bench	Kinematic + EMG
Kim (2018) [18]	Subjective	SUS	Daily living scenarios	User-reported satisfaction

**Table 5:** Experimental conditions (driving conditions)–course driving.

Guillon et al. (2015) [27]	Algood et al. [37,39]
5 m long concrete ramp with a 5% upward slope 90° turn 5 m long concrete ramp with a 10% upward slope 180° turn on a concrete landing, return down the 10% and 5% ramps 90° turn 5 m long path covered with cobblestones 180° turn Crossing over a 3 cm high door threshold 5 m long concrete ramp with a 15% upward slope 448 m long concrete ramp with a 6% upward slope 7 cm high curb to be climbed up and then down	Propel down a 61 m tiled hallway Open and propel through a 1.04 m wide door that opens away from the participants and has an accessible handle Propel across a 2.4 m long strip of high-pile carpet Propel across a 2.4 m long dimple strip (guide strip for individuals with visual impairments, where the dimples are 0.65 cm high) Propel up and down a 4° sloped ramp that is 6.7 m long Propel over a sinusoidal bump that is 50 mm high (simulating a speed bump) Propel up a 1.2 m long, 7.3° curb cut Propel up and down a 5.1 cm high curb Propel over a metal door threshold that is 2.54 cm high and 0.91 m wide Propel across a deck surface that is 15.2 m long Maneuver through a tight bathroom setup, propelling up to a toilet, sink, and bathtub to a spot where they would potentially use that bathroom fixture (e.g., point where they would use the sink) (each fixture was treated as a separate obstacle) Pick up a can of soup located 30.5 cm from the edge of a 0.81 m high cabinet, then propel the wheelchair 2.4 m and place the can in a similar location on another cabinet Turn on a kitchen faucet, using the accessible handle located 0.97 m from the floor and 45 cm from the front of the cabinet Maneuver into a simulated bus docking space (0.761.20 m)

**Table 6:** Comparison of subjective and objective usability outcomes.

Study	Subjective Tool	Objective Tool	User Group	Main Findings
Kim (2018) [18]	SUS	-	SCI	Usability high but varied by user type
Deems-Dluhy (2017) [22]	QUEST 2.0	Motion Capture	SCI	Device ease of use differed by prototype
Guillon (2015) [27]	VAS Satisfaction	Physiological Sensor	SCI	Heart rate and satisfaction varied across environments
Walford (2021) [31]	WheelCon-SF	SmartWheel	SCI	Pain patterns not strongly linked to propulsion technique

**Table 7:** Motion analysis data—sensor placement.

	First Author (Year)	Anatomical Landmarks	Wheelchair Landmarks
1	O. Jahanian (2022) [2]	Spinal process (C7), xiphoid process, suprasternal notch, acromion process, acromial angle, trigonum spinae, scapular spine, acromion angle, scapula inferior angle, coracoid process, humerus, olecranon process, radial styloid, ulnar styloid, third metacarpal, and fifth metacarpal	Top and bottom corners of wheelchair back, and the center of wheel hub
2	Lalumiere et al. (2013) [21]	Twenty-three skin-fixed markers on head, AC joint, thorax, shoulder blade, sternum, arm, elbow joint, forearm, wrist joint, and hand	Four markers
3	C.J. Newsam (1999) [24]	Manubrium, xiphoid process, spinous process of the seventh cervical vertebrae, greater tubercle of the humerus, mid-humerus, lateral epicondyle, medial epicondyle, proximal ulna, distal ulna, ulnar styloid process, radial styloid process, lateral border of the fifth metacarpal, and head of the third metacarpal	
4	K. Kulig (2001) [25]	Selected landmarks of the right upper extremity and trunk (upper arm, forearm, hand, and trunk)	The two axes of the wheel
5	R. Price (2007) [28]	Temporomandibular joint (bilateral), C7 and T2 vertebrae, sternal notch, along with unilateral markers on the nondominant side: acromion, elbow lateral epicondyle, olecranon, radial, and ulnar styloids and third metacarpal joint	The hub and a spoke of the SmartWheel on the non-dominant side
6	P. Cavallone [30] (2022)	Nexus Plug-in Gait marker set (protocol to model only the trunk, arm, and forearm segments)	
7	S.L. Walford (2021) [31]	Fifteen active markers were placed on body segment landmarks (Lighthall-Haubert et al., 2009 [35])	The right wheel
8	L. Lighthall-Haubert (2009) [35]	Manubrium, xiphoid process, T3 and T10 spinous processes, and the dorsal third metacarpal and medial fifth metacarpal phalangeal joints	Three markers attached to the spokes of the right wheel
9	G. Desroches (2009) [45]	Sternal notch, processus xiphoideus, C7, T8, angulus acromialis, superior part of the acromion, acromioclavicular joint, deltoid tuberosity, lateral and medial epicondyles, superior part of the forearm, ulnar and radial styloid processes, 2nd and 5th metacarpal heads, and one on the left acromial process	Upper and lower right corners of the backrest, upper and lower left corners of the backrest, right and left extremities of the seat, and one on each wheel center.
10	S.D. Algood (2004) [37]	Temporomandibular joint, acromion process, olecranon, lateral epicondyle, radial styloid, ulnar styloid, third metacarpophalangeal joint (MPJ), fifth MPJ, and three markers for the trunk	
11	J.L. Mercer [38] (2006)	Third metacarpophalangeal joint, radial styloid, ulnar styloid, lateral epicondyle, and acromion	Wheel hubs
12	M. L. Boninger (2004) [39]	Lateral epicondyle, ulnar styloid, radial styloid, and third metacarpophalangeal joint (MCJ)	
13	Y.S. Yang (2006) [41]	Upper body (acromion process, lateral epicondyle, and head of the third metacarpal) and hip (greater trochanter)	
14	W.M. Richter (2007) [42]	Third metacarpophalangeal joint (MPJ)—right hand	
15	J.L. Collinger (2008) [43]	Third metacarpophalangeal joint, radial styloid, ulnar styloid, lateral epicondyle, acromion, sternal notch, C7 vertebrae, T3 vertebrae, and greater trochanter	
16	N. Louis (2010) [44]	Clavicle, arm, forearm, hand, trunk (thorax and pelvis), and thigh	Fourteen reflective markers
17	D.J.J. Bregman (2008) [15]	Right side of the participants’ body (thorax, upper arm, forearm, and hand) and elbow (medial epicondyle)	Active markers

**Table 8:** Comparative summary of propulsion technologies.

Propulsion Type	Example Study (Year)	Evaluation Method	Key Objective Findings	Key Subjective Feedback
Conventional Pushrim	Kim (2018) [18]	SUS (subjective)	Efficient for basic mobility; high physical demand	High usability scores; learnability issues
PAPAW (Power-Assist)	Algood (2004, 2005) [37,40]	Motion + physiological	Reduced muscle load, HR, energy cost	Positive feedback on ease of use, comfort
Lever-based	Jahanian (2022) [2]	EMG + SMARTWheel	Reduced shoulder loading in low-gear condition	Preference for lower effort propulsion
Pulley–Cable (Rowing)	Cavallone (2022) [30]	Motion + EMG	Greater range of elbow motion; higher speed possible	Reported less shoulder fatigue
EHDM (Hand-Drive)	Zukowski (2014) [33]	VO_2_ + velocity measures	Lower efficiency than pushrim MWC	Needs gearing improvement

## Data Availability

The data that support the findings of this study are available from the corresponding author (J.P.) upon reasonable request.

## Supplementary Materials

The following supporting information can be downloaded at: https://www.mdpi.com/article/10.3390/jcm14093184/s1, Table S1: Methodological Quality Assessment of Included Studies. References [48,49,50,51,52,53,54,55,56,57] are cited in the supplementary materials.
