# Supplementary material for: Systematic Review of Usability Tests for Manual Wheelchairs: Enhancing Mobility and Reducing Shoulder Injuries in Individuals with Spinal Cord Injuries
Source: J Clin Med. 2025 May 4;14(9):3184. doi: 10.3390/jcm14093184 (PMC12072826; doi:10.3390/jcm14093184)
Supplement: Supplementary file 1 [file jcm-14-03184-s001.zip › jcm-3530965-supplementary.pdf]

Supplementary Table

Table S1. Methodological Quality Assessment of Included Studies

First Author (Year)	Objectives Clarity	Participant Description	Outcome Measure Validity	Usability Test Clarity	Analysis Rigor	Overall Rating
Kim (2018)	✗	✗	✓	✗	✗	Low
Omid Jahanian (2022a)	✓	✓	✗	✓	✗	Moderate
Omid Jahanian (2022b)	✓	✓	✗	✓	✗	Moderate
Mathieu Lalumiere (2013)	✓	✓	Partial	✓	✗	Moderate
Susan L. Deems-Dluhy (2017)	✓	✓	✓	✓	✗	High
Angel Gil-Agudo (2010)	✓	✓	✗	✓	✗	Moderate
Craig J. Newsam (1999)	✓	✓	✗	✓	✗	Moderate
Kornelia Kulig (2001)	✗	✓	✗	✓	✗	Moderate
Ursina Arnet (2024)	✓	✓	✗	✓	✗	Moderate
Bruno Guillon (2015)	✓	✓	Partial	✓	✗	Moderate
Robert Price (2007)	✓	✓	✗	✓	✗	Moderate
Mark S. Nash (2008)	✗	✓	✓	✓	✗	Moderate
Paride Cavallone (2022)	✓	Partial	✗	✓	✗	Moderate
Shelby L. Walford (2021)	✗	✓	✗	✓	✗	Moderate

Beth A. Cloud (2017)	✓	✓	✗	✓	✗	Moderate
Lisa A. Zukowski (2014)	✓	✓	✗	✗	✗	Moderate
Rachel E. Cowan (2008)	✓	✓	✗	✓	✗	Moderate
Lisa Lighthall-Haubert (2009)	✓	✓	✓	✓	✗	High
Brittany Bickelhaupt (2018)	✗	✗	✓	✓	✗	Moderate
S. David Algood (2004)	✗	✓	✗	✓	✗	Moderate
Jennifer L. Mercer (2006)	✗	✓	✗	✓	✗	Moderate
Michael L. Boninger (2004)	✓	✓	✗	✓	✗	Moderate
S. David Algood (2005)	✓	✓	✓	✓	✗	High
Yu-Sheng Yang (2006)	✗	✗	✓	✓	✗	Moderate
W. Mark Richter (2007)	✓	✓	✗	✓	✗	Moderate
Jennifer L. Collinger (2008)	✓	✓	✗	✓	✗	Moderate
Rory A. Cooper (2001)	✓	✓	✗	✓	✗	Moderate
N. Louis (2010)	✗	✓	✓	✓	✗	Moderate
D.J.J. Bregman (2008)	✗	✓	✗	✗	✗	Low
Johanne Mattie (2020)	✓	✓	✓	✓	✗	High
